# Supplementary material for: Environmental Noise Exposure and Sleep Habits among Children in a Cohort from Northern Spain
Source: Int J Environ Res Public Health. 2022 Dec 6;19(23):16321. doi: 10.3390/ijerph192316321 (PMC9738338; doi:10.3390/ijerph192316321)
Supplement: Supplementary file 1 [file ijerph-19-16321-s001.zip › ijerph-2031333-supplementary.pdf]

Appendix SA: Ad hoc q*uestionnaires used in the study. Original questionnaires were in Spanish or Basque.*

SA1. Questionnaire used to asses perceived sleep variables.

SLEEP HABITS

Instructions: The following questions are related to your child's sleep habits. Try to remember what has happened IN THE PAST YEAR as accurately as possible for most days and nights. Please answer all questions.

	a. Weekdays	b. Non-working days or weekends
**81. At what time does he/she usually go to bed at night?**	____h ____min	____h ____min
**82. Considering that he/she can stay awake for some time in bed (e.g., reading), at what time does he/she turn off the light and are ready to go to sleep?**	____h ____min	____h ____min
**83. How many minutes does it usually take him/her to fall asleep each night?**	____min	____min
**84. At what time does he/she usually get up in the morning?**	____h ____min	____h ____min

SA2. Stressful family events questionnaire.

STRESSFUL FAMILY EVENTS

Instructions: Some of the following questions refer to events that are very important to the child. Please indicate if the following events have taken place throughout your child's life.

	Yes	No
**146. Residence change**	☐	☐
**147. School change**	☐	☐
**148. Parents' separation or divorce**	☐	☐
**149. Death of a family member**	☐	☐
**150. Long-term hospitalization**	☐	☐

SA3. Questionnaire about physical activity. Example of the questionnaire used is presented: Apart from the 4 exercises presented in the example, other 27 are also presented: Football, swimming, riding a horse, riding the bicycle, running, playing tennis, etc.

PHYSICAL ACTIVITIES

Instructions: The following questions are related to your child's physical activities. **In a typical week, how many minutes does your child spend doing the following physical activities per day on a tipical week (excluding school trips)?** *Please, explain for each exercise the day of the week and time he/she spend doing it.*

EXAMPLE: Your child does the following physical exercises: (1) Basketball training on Monday and Wednesday during 90 minutes and on Saturday a 60 minutes long match. (2) On Saturday she/he goes for a walk in the mountain (3) Dances on Tuesday and Thursday during 30 min, (4) on Tuesday and Thursday plays active videogames in the Wii, 60 minutes each day

Exercise	Week day						
	Monday (minutes)	Tuesday (minutes)	Wednesday (minutes)	Thursday (minutes)	Friday (minutes)	Saturday (minutes)	Sunday (minutes)
Go for a walk in the mountain						120	
Basketball	90		90			60	
Dance		30		30			
Play active videogames/Wii		60		60			

Appendix SB: *Descriptive statistics*

Table SB1. Descriptive statistics of the numerical covariables present in the study

Variable	N	Mean	SD	Min	Q1	Median	Q3	Max	IQR
Age	377	10.8	0.2	10.2	10.7	10.8	11.0	11.8	0.3
Maternal age	375	43.47	3.19	35.49	41.25	42.87	45.54	54.31	4.29
Number of recorded nights	135	6.16	1.49	1.00	6.00	7.00	7.00	7.00	1.00
Hair cortisol concentration (pg/mg)	346	11.13	13.10	1.25	7.21	8.62	11.15	160.00	152.79
BMI (kg/m^2)	374	18.9	3.2	10.7	16.8	18.4	20.50	30.6	3.7
Psychological well-being (KIDSCREEN-27)	377	2.5	1.2	0.6	1.8	2.5	3.6	4.9	1.9
School environment (KIDSCREEN-27)	377	2.7	1.4	0.2	2.0	2.6	3.3	4.7	1.4
Personal psychological skills (SDQ)	372	13.3	3.9	6.0	10.0	13.0	15.0	27.0	5.0
Objective Moderate/Vigorous physical activity (Actigraph, min/day)	135	82.7	29.5	25.2	62.1	76.8	100.3	167.9	38.3
Subjective Moderate/Vigorous physical activity (Questionnaire, min/day)	374	112.6	29.5	8.6	68.6	98.5	137.9	462.9	69.3

Table SB2.Sleep habits means for categorical variable groups. For each group, sample size (N), frequencies and nocturnal sleep/sleep period and sleep latency means are shown both parental reported and measured by actigraph.

		**Measured by Actigraph**		**Parental reprted**	
		**Nocturnal Sleep (h)** ***(SD)***	**Sleep Latency (h)** ***(SD)***	**Sleep period (h)** ***(SD)***	**Sleep Latency (h)** ***(SD)***
Sex	Male	7.81 *(0.62)*	1.39 *(0.40)*	9.42 *(0.62)*	0.76 *(0.35)*
	Female	7.67 *(0.54)*	1.41 *(0.46)*	9.33 *(0.50)*	0.93 *(0.41)*
Neighbourhood SES	I Higher	7.96 *(0.57)*	1.47 (0.29)	9.51 *(0.59)*	0.78 *(0.43)*
	II	7.75 *(0.64)*	1.32 (0.41)	9.38 *(0.46)*	0.87 *(0.38)*
	III	7.66 *(0.62)*	1.48 (0.49)	9.31 *(0.47)*	0.86 *(0.42)*
	IV	7.75 *(0.50)*	1.35 (0.30)	9.43 *(0.47)*	0.82 *(0.36)*
	V Lower	8.08 *(0.45)*	1.74 (0.91)	9.38 *(0.43)*	0.84 *(0.39)*
Number of stressful events	0	7.67 *(0.62)*	1.43 *(0.46)*	9.36 *(0.46)*	0.85 *(0.40)*
	1	7.82 *(0.59)*	1.38 *(0.43)*	9.40 *(0.51)*	0.89 *(0.42)*
	2 or more	7.80 *(0.53)*	1.30 *(0.35)*	9.36 *(0.46)*	0.78 *(0.30)*
Smartphone (computer, tablet) usage at bedtime (SCTUB)	No	7.69 *(0.53)*	1.46 *(0.47)*	9.43 *(0.48)*	0.85 *(0.43)*
	Yes	7.74 *(0.62)*	1.38 *(0.43)*	9.35 *(0.47)*	0.85 *(0.39)*
Smartphone (computer, tablet) usage during the night (SCTUDN)	No	7.74 *(0.60)*	1.39 *(0.44)*	9.39 *(0.48)*	0.85 *(0.40)*
	Yes	7.64 *(0.63)*	1.40 *(0.44)*	9.30 *(0.46)*	0.86 *(0.32)*

Victim propensity	No	7.73 *(0.60)*	1.42 *(0.44)*	9.36 *(0.47)*	0.86 *(0.39)*
	Yes	7.80 *(0.66)*	1.17 *(0.35)*	9.48 *(0.47)*	0.78 *(0.39)*
Bully/victim	No	7.72 *(0.60)*	1.40 *(0.43)*	9.37 *(0.48)*	0.85 *(0.39)*
	Yes	8.14 (0.30)	1.23 *(0.80)*	9.50 (0.26)	0.91 *(0.17)*

Appendix SC: *Correlation table*

Table SC1. Correlations between response variables

Variable pair		t-value	d.f.	p-value	Pearson corr. r	95% C.I. for r
Time In Bed	Nocturnal Sleep	13.136	133	<0.001***	0.75	(0.667, 0.817)
Time In Bed	Sleep Latency	5.53	133	<0.001***	0.43	(0.284, 0.560)
Time In Bed	Sleep Efficiency	-2.26	133	0.012**	-0.21	(-0.369, -0.047)
Time In Bed	Diurnal Rest	-3.96	133	<0.001***	-0.325	(-0.468, -0.165)
Nocturnal Sleep	Sleep Latency	-3.233	133	0.002**	-0.27	(-0.419, -0.106)
Nocturnal Sleep	Sleep Efficiency	6.318	133	<0.001***	0.48	(-0.339, -0.600)
Nocturnal Sleep	Diurnal Rest	-5.28	133	<0.001***	-0.42	(-0.547, -0.266)
Sleep Latency	Sleep Efficiency	-45.14	133	<0.001***	-0.969	(-0.977, -0.956)
Sleep Latency	Diurnal Rest	1.095	133	0.27	0.09	(-0.07, -0.26)
Sleep Efficiency	Diurnal Rest	-2.37	133	0.019**	-0.2	(-0.35, -0.033)
Reported Time In Bed	Reported Sleep Period	14.24	365	<0.001***	0.6	(0.53, 0.65)
Reported Time In Bed	Reported Sleep Latency	6.32	364	<0.001***	0.31	(0,219, 0.404)
Reported Time In Bed	Reported Sleep Efficiency	-4.54	364	<0.001***	-0.23	(-0.33, -0.132)
Reported Sleep Period	Reported Sleep Latency	-12.9	364	<0.001***	-0.56	(-0.627, -0.486)
Reported Sleep Period	Reported Sleep Efficiency	15.43	364	<0.001***	0.629	(0.562, 0.688)
Reported Sleep Efficiency	Reported Sleep Latency	-204.0	364	<0.001***	-0.996	(-0.9964, -0.9946)
Time In Bed	Reported Time In Bed	0.462	131	0.645	0.04	(-0.13, 0.209)
Nocturnal Sleep	Reported Sleep Period	-0.106	132	0.915	-0.009	(-0.179, 0.161)
Sleep Latency	Reported Sleep Latency	0.904	130	0.368	0.08	(-0.09, 0.247)
Sleep Efficiency	Reported Sleep Efficiency	0.76	130	0.45	0.067	(-0.116, 0.235)

Note: **p<0.01; ***p<0.001

Appendix SD: *DAGgity code for the current model.*

```
dag {

bb="-0.5,-0.5,0.5,0.5"

"Cortisol levels" [pos="0.154,-0.241"]

"Environmental Noise" [exposure,pos="-0.380,0.188"]

"Family legacy" [latent,pos="-0.275,0.352"]

"Historical legacy" [latent,pos="-0.424,-0.439"]

"Physical activity" [pos="0.076,0.410"]

"Psychological well-being" [pos="0.072,0.024"]

"School environment" [pos="-0.063,-0.450"]

"Sleep habits" [outcome,pos="0.421,0.182"]

"Smartphone usage" [pos="0.445,-0.150"]

"Stressful events" [pos="0.396,-0.335"]

"Urban Planning" [latent,pos="-0.430,-0.127"]

BMI [pos="0.228,0.244"]

Bullying [pos="-0.224,-0.217"]

SES [pos="-0.135,0.106"]

Sex [pos="0.302,-0.447"]

"Cortisol levels" -> "Sleep habits"

"Environmental Noise" -> "Cortisol levels"

"Environmental Noise" -> "Sleep habits"

"Family legacy" -> SES

"Historical legacy" -> "Urban Planning"

"Physical activity" -> "Cortisol levels"

"Physical activity" -> "Sleep habits"

"Physical activity" -> BMI

"Psychological well-being" -> "Cortisol levels"

"Psychological well-being" -> "Sleep habits"

"School environment" -> "Psychological well-being"

"School environment" -> Bullying

"Smartphone usage" -> "Sleep habits"

"Stressful events" -> "Cortisol levels"

"Stressful events" -> "Psychological well-being"

"Stressful events" -> "Sleep habits"
```

```
"Urban Planning" -> "Environmental Noise"

BMI -> "Cortisol levels"

BMI -> Bullying

Bullying -> "Cortisol levels"

Bullying -> "Psychological well-being"

Bullying -> "Sleep habits"

SES -> "Cortisol levels"

SES -> "Environmental Noise"

SES -> "Physical activity"

SES -> "Psychological well-being"

SES -> "Sleep habits"

SES -> BMI

SES -> Bullying

Sex -> "Cortisol levels"

Sex -> "Physical activity"

Sex -> "Sleep habits"

Sex -> Bullying

}
```

Appendix SE: *Testable implications for the actigraphic data.*

Table SE1. Testable implications for actigraphic data. Data from the 135 children who wore the actigraph were used; with information being provided in relation to sleep habits and physical activity. Statistical test used depend on nature of tested variables, and therefore, T-value, F-value, Kruskal-Wallis, Chi-sq, Welch´s T-values are reported Note: BMI, Body Mass Index; SES, Sociodemographic status; SDQ, Strengths and Difficulties Questionnaire; SCTUB, Smartphone computer and tablet use at bedtime; SCTUDN, Smartphone computer and tablet use during the night; School environment and Psychological well-being are Kidscreen-27 subscales.

Testable implication	t-value	d.f.	p-value	Pearson corr. r	95% C.I. for r
Cortisol levels ⊥ School environment | BMI, Bullying, Psychological well-being, SES, Sex, Stressful events	1.403	119	0.163	0.127	(-0.052, 0.299)
Evening Environmental Noise ⊥ Physical activity | SES	0.377	125	0.707	0.034	(-0.141. 0.207)
Evening Environmental Noise ⊥ Psychological well-being | SES	-0.588	125	0.558	-0.052	(-0.224, 0.123)
Evening Environmental Noise ⊥ SDQ| SES	-0.983	125	0.327	-0.09	(-0.26, 0.088)
Evening Environmental Noise ⊥ School environment	0.658	125	0.511	0.058	(-0.116- 0.231)
Evening Environmental Noise ⊥ BMI | SES	0.368	125	0.714	-0.03	(-0.206, 0.142)
Evening Environmental Noise ⊥ Victim (Bullying) | SES	0.7535	125	0.456	0.067	(-0.108, 0.239)
Evening Environmental Noise ⊥ Bully/Victim (Bullying) | SES	0.687	125	0.4932	0.061	(-0.114, 0.233)
Nocturnal Environmental Noise ⊥ Physical activity | SES	-0.005	125	0.998	-0.0004	(-0.175, 0.173)
Nocturnal Environmental Noise ⊥ Psychological well-being | SES	-0.015	125	0.989	-0.001	(-0.175, 0.173)
Nocturnal Environmental Noise ⊥ SDQ | SES	-0.674	125	0.501	-0.06	(-0.232, 0.115)
Nocturnal Environmental Noise ⊥ School environment	0.804	124	0.423	0.072	(-0.104, 0.244)
Nocturnal Environmental Noise ⊥ BMI | SES	-0.791	125	0.430	-0.07	(-0.242, 0.105)
Nocturnal Environmental Noise ⊥ Victim (Bullying) | SES	-0.127	125	0.899	-0.011	(-0.185, 0.163)
Nocturnal Environmental Noise ⊥ Bully/Victim (Bullying) | SES	0.035	125	0.972	0.003	(-0.17, 0.177)
BMI ⊥ Sex | Physical activity, SES	1.293	133	0.198	0.111	(-0.059, 0.275)
Physical activity ⊥ Psychological well-being | Bullying, SES, School environment	0.845	133	0.399	0.073	(-0.01, 0.239)
Physical activity ⊥ SDQ| Bullying, SES, School environment	-0.263	133	0.814	-0.02	(-0.189, 0.149)
Physical activity ⊥ Psychological well-being | BMI, SES, Sex	0.541	133	0.59	0.047	(-0.123, 0.214)
Physical activity ⊥ SDQ| BMI, SES, Sex	-0.487	133	0.627	-0.042	(-0.210, 0.128)
Physical activity ⊥ School environment	-0.515	133	0.607	-0.045	(-0.212, 0.125)
Physical activity ⊥ Victim (Bullying) | BMI, SES, Sex	-2.044	133	0.043*	-0.17	(-0.33, 0.006)
Physical activity ⊥ Bully/Victim (Bullying)| BMI, SES, Sex	-0.676	133	0.5	-0.059	(-0225, 0.111)
Psychological well-being ⊥ BMI | Bullying, SES, School environment	-1.225	133	0.223	-0.106	(-0.269, 0.064)
Psychological well-being ⊥ Sex | Bullying, SES, and School environment	-0.211	133	0.833	-0.018	(-0.187, 0.151)
SDQ ⊥ BMI | Bullying, SES, School environment	-0.442	133	0.659	-0.038	(-0.206, 0.132)
SDQ ⊥ Sex | Bullying, SES, and School environment	1.135	133	0.259	0.098	(-0.072, 0.263)
School environment ⊥ Time In Bed| Bullying, Cortisol levels, Environmental Noise, Physical activity, Psychological well-being, SES, Sex, Stressful events	-0.499	112	0.619	-0.047	(-0.229, 0.138)
School environment ⊥ Nocturnal Sleep | Bullying, Cortisol levels, Environmental Noise, Physical activity, Psychological well-being, SES, Sex, Stressful events	-0.01	112	0.989	-0.001	(-0.185, 0.183)

School environment ⊥ Sleep Latency | Bullying, Cortisol levels, Environmental Noise, Physical activity, Psychological well-being, SES, Sex, Stressful events	-0.726	112	0.482	-0.067	(-0.247, 0.119)
School environment ⊥ Sleep Efficiency| Bullying, Cortisol levels, Environmental Noise, Physical activity, Psychological well-being, SES, Sex, Stressful events	0.587	112	0.559	0.055	(-0.13, 0.239)
School environment ⊥ Diurnal Rest| Bullying, Cortisol levels, Environmental Noise, Physical activity, Psychological well-being, SES, Sex, Stressful events	-0.247	112	0.806	-0.02	(-0.206, -0.161)
School environment ⊥ Time In Bed| BMI, Bullying, Psychological well-being, SES, Sex, Stressful events	-0.576	133	0.57	-0.049	(0.217, 0,120)
School environment ⊥ Nocturnal Sleep| BMI, Bullying, Psychological well-being, SES, Sex, Stressful events	0.072	133	0.943	0.006	(-0.163, 0.175)
School environment ⊥ Sleep Latency| BMI, Bullying, Psychological well-being, SES, Sex, Stressful events	-0.935	133	0.351	-0.081	(-0.246, 0.089)
School environment ⊥ Sleep Efficiency| BMI, Bullying, Psychological well-being, SES, Sex, Stressful events	0.802	133	0.424	0.069	(-0.101, 0.236)
School environment ⊥ Diurnal Rest| BMI, Bullying, Psychological well-being, SES, Sex, Stressful events	-0.542	133	0.589	-0.047	(-0.214, -0.123)
School environment ⊥ BMI	0.296	133	0.768	0.026	(-0.144, 0.194)
Time In Bed ⊥ BMI | Bullying, Cortisol levels, Environmental Noise, Physical activity, Psychological well-being, SES, Sex, Stressful events	-0.368	112	0.713	-0.034	(-0.217, 0.150)
Nocturnal Sleep ⊥ BMI | Bullying, Cortisol levels, Environmental Noise, Physical activity, Psychological well-being, SES, Sex, Stressful events	-0.595	112	0.553	-0.06	(-0.237, 0.129)
Sleep Latency ⊥ BMI | Bullying, Cortisol levels, Environmental Noise, Physical activity, Psychological well-being, SES, Sex, Stressful events	0.28	112	0.778	0.026	(-0.158, 0.209)
Sleep Efficiency ⊥ BMI | Bullying, Cortisol levels, Environmental Noise, Physical activity, Psychological well-being, SES, Sex, Stressful events	-0.448	112	0.655	-0.042	(-0.224, 0.143)
Diurnal Rest ⊥BMI | Bullying, Cortisol levels, Environmental Noise, Physical activity, Psychological well-being, SES, Sex, Stressful events	0.162	112	0.871	0.015	(-0.169, 0.199)

**Testable implication**	**F-value**	**d.f.**	**p-value**	**R-squared**	**Adjusted R-squared**
Cortisol levels ⊥ SCTUB	1.357	1 and 119	0.246	0.01	0.002
Cortisol levels ⊥ SCTUDN	1.325	1 and 119	0.25	0.011	0.002
Evening Environmental Noise ⊥ SCTUB	0.740	1 and 125	0.391	0.005	-0.002
Evening Environmental Noise ⊥ SCTUDN	0.005	1 and 125	0.946	0.0007	-0.007
Evening Environmental Noise ⊥ Stressful events	2.125	2 and 124	0.121	0.034	0.018
Nocturnal Environmental Noise ⊥ SCTUB	0.004	1 and 125	0.95	0.003	-0.008
Nocturnal Environmental Noise ⊥ SCTUDN	0.566	1 and 125	0.453	0.005	-0.003
Nocturnal Environmental Noise ⊥ Stressful events	0.945	2 and 124	0.391	0.015	-0.0009
Physical activity ⊥ SCTUB	2.67	1 and 133	0.1	0.02	0.012
Physical activity ⊥ SCTUDN	3.13	1 and 133	0.08	0.02	0.015
Physical activity ⊥ Stressful events	0.229	2 and 132	0.795	0.003	-0.01
SCTUB⊥BMI	0.04	1 and 133	0.836	0.0003	-0.007
SCTUDN⊥BMI	0.472	1 and 133	0.493	0.004	-0.004
Stressful events ⊥ BMI	0.328	1 and 133	0.569	0.002	-0.005

**Testable implication**	**t-value**	**d.f.**	**p-value**	**R-squared**	**Adjusted R-squared**
Evening Environmental Noise ⊥ Sex	0.729	108.68	0.474	0.004	-0.003
Nocturnal Environmental Noise ⊥ Sex	1.582	123.48	0.116	0.018	0.01
SDQ ⊥ SCTUB	0.02	67.54	0.98	0.0001	-0.007

SDQ ⊥ SCTUDN	0.858	21.76	0.4	0.017	0.009

Testable implication	K.W chi.sq	d.f.	p-value
School environment ⊥ Stressful events	1.357	2	0.507
School environment ⊥ SES	2.96	4	0.564
**Testable implication**	**W**	**p-value**	
School environment ⊥ Sex	2389.5	0.42	
Psychological well-being ⊥ SCTUB	1652	0.985	
Psychological well-being ⊥ SCTUDN	1094	0.791	
School environment ⊥ SCTUB	1628.5	0.92	
School environment ⊥ SCTUDN	1199.5	0.336	
**Testable implication**	**χ^2**	**d.f.**	**p-value**
SCTUB ⊥ Victim (Bullying)	0.361	1	0.731
SCTUB ⊥ Bully/Victim (Bullying)	0.63	1	0.42
SCTUB ⊥ SES	4.236	4	0.375
SCTUB ⊥ Sex	4.69	1	0.045*
SCTUB ⊥ Stressful events	3.346	2	0.19
SCTUDN ⊥ Stressful events	0.479	2	0.82
SCTUDN ⊥ Victim (Bullying)	4.56	1	0.06
SCTUDN ⊥ Bully/Victim (Bullying)	0.31	1	0.576
SCTUDN ⊥ SES	3.11	4	0.539
SCTUDN ⊥ Sex	5.5	1	0.06
Stressful events ⊥ Victim (Bullying)	0.958	2	0.619
Stressful events ⊥ Bully/Victim (Bullying)	1.529	2	0.668
Stressful events ⊥ SES	6.197	4	0.185
Stressful events ⊥ Sex	2.915	2	0.233
SES ⊥ Sex	0.155	2	0.926

Note: *p<0.05

Appendix SF*: Testable implications for the parental reported data.*

Table SF1. Testable implications for subjective data. Data from all 377 children was used, with information regarding sleep habits and physical activity being estimated from questionnaires completed by parents. Statistical test used depend on nature of tested variables, and therefore, T-value, F-value, Kruskal-Wallis, Chi-sq, Welch´s T-values are reported. Note: BMI, Body Mass Index; SES, Sociodemographic status; SDQ, Strengths and Difficulties Questionnaire; SCTUB, Smartphone computer and tablet use at bedtime; SCTUDN, Smartphone computer and tablet use during night-time; School environment and Psychological well-being are Kidscreen-27 subscales.

Testable implication	t-value	d.f.	p-value	Pearson corr. r	95% C.I. for r
Cortisol levels ⊥ School environment | BMI, Bullying, Psychological well-being, SES, Sex, Stressful events	0.545	342	0.586	0.029	(-0.076, 0.135)
Evening Environmental Noise ⊥ Physical activity | SES	-0.674	325	0.5	-0.037	(-0.145, 0.071)
Evening Environmental Noise ⊥ Psychological well-being | SES	-0.471	326	0.638	-0.026	(-0.133, 0.082)
Evening Environmental Noise ⊥ SDQ| SES	-0.328	324	0.743	-0.018	(-0.127, 0.09)
Evening Environmental Noise ⊥ School environment	0.699	326	0.485	0.039	(-0.07- 0.146)
Evening Environmental Noise ⊥ BMI | SES	-1.84	325	0.1	0.067	(-0.208, 0.01)
Evening Environmental Noise ⊥ Victim (Bullying) | SES	0.95	326	0.343	0.053	(-0.056, 0.16)
Evening Environmental Noise ⊥ Bully/Victim (Bullying) | SES	1.279	326	0.202	0.071	(-0.038, 0.178)
Nocturnal Environmental Noise ⊥ Physical activity | SES	-0.473	325	0.636	-0.026	(-0.134, 0.08)
Nocturnal Environmental Noise ⊥ Psychological well-being | SES	0.422	326	0.673	0.023	(-0.085, 0.131)
Nocturnal Environmental Noise ⊥ SDQ | SES	0.035	324	0.972	0.002	(-0.107, 0.111)
Nocturnal Environmental Noise ⊥ School environment	1.343	326	0.18	0.074	(-0.034, 0.181)
Nocturnal Environmental Noise ⊥ BMI | SES	-1.809	325	0.07	-0.01	(-0.21, 0.009)
Nocturnal Environmental Noise ⊥ Victim (Bullying) | SES	0.545	326	0.586	0.03	(-0.078, 0.138)
Nocturnal Environmental Noise ⊥ Bully/Victim (Bullying) | SES	1.382	326	0.17	0.076	(-0,03, 0.183)
BMI ⊥ Sex | Physical activity, SES	0.895	372	0.372	0.046	(-0.055, 0.147)
Physical activity ⊥ Psychological well-being | Bullying, SES, School environment	1.252	371	0.21	0.065	(-0.037, 0.165)
Physical activity ⊥ SDQ| Bullying, SES, School environment	-0.407	368	0.684	-0.021	(-0.123, 0.081)
Physical activity ⊥ Psychological well-being | BMI, SES, Sex	0.53	372	0.596	0.027	(-0.07, 0.128)
Physical activity ⊥ SDQ| BMI, SES, Sex	-0.855	369	0.393	-0.044	(-0.146, 0.057)
Physical activity ⊥ School environment	-1.292	372	0.197	-0.067	(-0.167, 0.035)
Physical activity ⊥ Victim (Bullying) | BMI, SES, Sex	0.636	371	0.525	0.033	(-0.068, 0.134)
Physical activity ⊥ Bully/Victim (Bullying)| BMI, SES, Sex	1.42	371	0.158	0.07	(-0.028, 0.174)
Psychological well-being ⊥ BMI | Bullying, SES, School environment	-0.494	371	0.622	-0.026	(-0.127, 0.076)
Psychological well-being ⊥ Sex | Bullying, SES, and School environment	-2.24	374	0.04*	-0.11	(-0.21, 0.014)

SDQ ⊥ BMI | Bullying, SES, School environment	0.958	368	0.339	0.05	(-0.05, 0.15)
SDQ⊥ Sex | Bullying, SES, and School environment	2.076	369	0.04*	0.1	(0.006, 0.207)
School environment ⊥ Reported Time In Bed| Bullying, Cortisol levels, Environmental Noise, Physical activity, Psychological well-being, SES, Sex, Stressful events	0.732	298	0.465	0.042	(-0.071, 0.155)
School environment ⊥ Reported Nocturnal Sleep | Bullying, Cortisol levels, Environmental Noise, Physical activity, Psychological well-being, SES, Sex, Stressful events	1.23	299	0.221	0.071	(-0.04, 0.18)
School environment ⊥ Reported Sleep Latency | Bullying, Cortisol levels, Environmental Noise, Physical activity, Psychological well-being, SES, Sex, Stressful events	-0.1222	296	0.223	-0.07	(-0.18, 0.04)
School environment ⊥ Reported Sleep Efficiency| Bullying, Cortisol levels, Environmental Noise, Physical activity, Psychological well-being, SES, Sex, Stressful events	1.256	296	0.21	0.072	(-0.04, 0.185)
School environment ⊥ Reported Time In Bed| BMI, Bullying, Psychological well-being, SES, Sex, Stressful events	1.057	365	0.29	0.055	(-0.09, 0.259)
School environment ⊥ Reported Nocturnal Sleep| BMI, Bullying, Psychological well-being, SES, Sex, Stressful events	1.235	367	0.22	0.064	(-0.038, 0.165)
School environment ⊥ Reported Sleep Latency| BMI, Bullying, Psychological well-being, SES, Sex, Stressful events	-0.936	362	0.35	-0.076	(-0.04, 0.05)
School environment ⊥ Reported Sleep Efficiency| BMI, Bullying, Psychological well-being, SES, Sex, Stressful events	0.996	362	0.32	0.052	(-0.05, 0.154)
School environment ⊥ BMI	-0.207	133	0.836	-0.011	(-0.112, 0.091)
Reported Time In Bed ⊥ BMI | Bullying, Cortisol levels, Environmental Noise, Physical activity, Psychological well-being, SES, Sex, Stressful events	-1.78	298	0.08	-0.1	(-0.213, -0.011)
Reported Nocturnal Sleep ⊥ BMI | Bullying, Cortisol levels, Environmental Noise, Physical activity, Psychological well-being, SES, Sex, Stressful events	-1.683	299	0.09	-0.01	(-0.20, 0.01)
Reported Sleep Latency ⊥ BMI | Bullying, Cortisol levels, Environmental Noise, Physical activity, Psychological well-being, SES, Sex, Stressful events	0.62	296	0.872	0.009	(-0.104, 0.123)
Reported Sleep Efficiency ⊥ BMI | Bullying, Cortisol levels, Environmental Noise, Physical activity, Psychological well-being, SES, Sex, Stressful events	-0.287	296	0.77	-0.017	(-0.13, 0.097)

**Testable implication**	**F-value**	**d.f.**	**p-value**	**R-squared**	**Adjusted R-squared**
Cortisol levels ⊥ SCTUB	0.356	1 and 344	0.474	0.001	-0,001
Cortisol levels ⊥ SCTUDN	0.137	1 and 344	0.711	0.0003	-0,002
Evening Environmental Noise ⊥ SCTUB	3.26	1 and 326	0.07	0.01	0,006
Evening Environmental Noise ⊥ SCTUDN	0.855	1 and 326	0.36	0.002	0,36
Evening Environmental Noise ⊥ Stressful events	0.511	2 and 325	0.6	0.003	-0,003
Nocturnal Environmental Noise ⊥ SCTUB	3.01	1 and 326	0.08	0.01	0,08
Nocturnal Environmental Noise ⊥ SCTUDN	0.76	1 and 326	0.38	0.002	-0,007

Nocturnal Environmental Noise ⊥ Stressful events	0.369	2 and 325	0.692	0.002	-0,003
Physical activity ⊥ SCTUB	0.11	1 and 372	0.74	0.0003	-0,002
Physical activity ⊥ SCTUDN	3.6	1 and 372	0.06	0.009	0,007
Physical activity ⊥ Stressful events	1.44	2 and 371	0.24	0.008	0,002
SCTUB ⊥ BMI	0.09	1 and 372	0.758	0.0002	-0,002
SCTUDN ⊥ BMI	1.63	1 and 372	0.203	0.004	0,001
Stressful events ⊥ BMI	1.41	2 and 371	0.246	0.007	0,002

**Testable implication**	**t-value**	**d.f.**	**p-value**	**R-squared**	**Adjusted R-squared**
Evening Environmental Noise ⊥ Sex	0.977	303.48	0.33	0.003	-0,0001
Nocturnal Environmental Noise ⊥ Sex	-0.363	320.01	0.72	0.0003	-0,002
SDQ ⊥ SCTUB	-0.923	138.65	0.36	0.002	-0,001
SDQ ⊥ SCTUDN	-1.26	60.5	0.21	0.006	0,003

**Testable implication**	**W**	**p-value**
School environment ⊥ Sex	20018	0.463
Psychological well-being ⊥ SCTUB	13854	0.1
Psychological well-being ⊥ SCTUDN	9214.5	0.21
School environment ⊥ SCTUB	13156	0.39
School environment ⊥ SCTUDN	8984	0.35

**Testable implication**	**K.W chi.sq**	**d.f.**	**p-value**
School environment ⊥ Stressful events	2.489	2	0.29
School environment ⊥ SES	1.31	4	0.86

**Testable implication**	**χ^2**	**d.f.**	**p-value**
SCTUB ⊥ Victim (Bullying)	0.66	1	0.417
SCTUB ⊥ Bully/Victim (Bullying)	0.28	1	0.595
SCTUB ⊥ SES	5.406	4	0.06
SCTUB ⊥ Sex	1.53	1	0.22
SCTUB ⊥ Stressful events	0.373	2	0.83
SCTUDN ⊥ Stressful events	0.58	2	0.75
SCTUDN ⊥ Victim (Bullying)	1.364	1	0.30
SCTUDN ⊥ Bully/Victim (Bullying)	0.283	1	0.96
SCTUDN ⊥ SES	2.13	4	0.71
SCTUDN ⊥ Sex	2.86	1	0.09
Stressful events ⊥ Victim (Bullying)	2.06	2	0.36
Stressful events ⊥ Bully/Victim (Bullying)	1.17	2	0.55
Stressful events ⊥ SES	6.72	8	0.567
Stressful events ⊥ Sex	1.53	2	0.47
SES ⊥ Sex	5.97	4	0.201

Note: *p<0.05

Appendix SG*:* Direct effects of evening and nocturnal environmental noise on sleep habits

Table SG1: Direct effect of evening and nocturnal environmental noise on sleep habits, measured by actigraph. Not standardized B-estimates and 95% confidence intervals are shown.

			Time In Bed		Nocturnal Sleep		Sleep Latency		Sleep Efficiency		Diurnal Rest	
Model 2[(a)]	*Direct effect*	*Evening Environmental Noise*	-0.0060	(-0.025, 0.014)	-0.0001	(-0.019, 0.019)	-0.0050	(-0.019, 0.08)	0.05	(-0.8, 0.2)	-0.0080	(-0.029, 0.011)
		Nocturnal Environmental Noise	-0.0180	(-0.038, 0.003)	-0.0120	(-0.03, 0.007)	-0.0060	(-0.02, 0.008)	0.03	(-0.1, 0.17)	-0.0001	(-0.02, 0.019)
Model 3 [(b)]	*Direct effect*	*Evening Environmental Noise*	-0.0020	(-0.022, 0.018)	0.0020	(-0.016, 0.021)	-0.0050	(-0.018, 0.008)	0.05	(-0.08, 0.2)	-0.0090	(-0.029, 0.012)
		Nocturnal Environmental Noise	-0.0170	(-0.038, 0.004)	-0.0110	(-0.03, 0.009)	-0.0060	(-0.02, 0.008)	0.03	(-0.1, 0.2)	-0.0001	(-0.022, 0.021)
Model 4 [(c)]	*Direct effect*	*Evening Environmental Noise*	-0.0050	(-0.024, 0.015)	0.0020	(-0.017, 0.020)	-0.0063	(-0.019, 0.006)	0.06	(-0.07, 0.2)	-0.0097	(-0.03, 0.01)
		Nocturnal Environmental Noise	-0.0180	(-0.039, 0.003)	-0.0120	(-0.03, 0.008)	-0.0060	(-0.02, 0.008)	0.04	(-0.1, 0.2)	-0.0001	(-0.022, 0.021)

(a) Adjusted for bully/victim propensity (bullying), hair cortisol level, objective physical activity, psychological well-being as measured by the Kidscreen 27, socioeconomic status, sex and number of stressful events.

(b) Adjusted for victim propensity (bullying), hair cortisol level, objective physical activity, psychological well-being as measured by the SDQ, socioeconomic status, sex and number of stressful events.

(c) Adjusted for bully/victim propensity (bullying), hair cortisol level, objective physical activity, psychological well-being as measured by the SDQ, socioeconomic status, sex and number of stressful events.

Table SG2: Direct effect of evening and nocturnal environmental noise on sleep habits, measured subjectively. Not standardized B-estimates and 95% confidence intervals are shown.

			Reported Time In Bed		Reported Nocturnal Sleep		Reported Sleep Latency		Reported Sleep Efficiency	
Model 2[(a)]	*Direct effect*	*Evening Environmental Noise*	0.0050	(-0.002, 0.013)	0.0080	(-0.001, 0.017)	-0.0020	(-0.01, 0.004)	0.03	(-0.04, 0.1)
		Nocturnal Environmental Noise	0.0050	(-0.004, 0.013)	0.0070	(-0.002, 0.016)	-0.0030	(-0.01, 0.005)	0.03	(-0.4, 0.1)
Model 3 [(b)]	*Direct effect*	*Evening Environmental Noise*	0.0050	(-0.002, 0.013)	0.0070	(-0.002, 0.016)	-0.0020	(-0.009, 0.005)	0.02	(-0.4, 0.09)
		Nocturnal Environmental Noise	0.0050	(-0.003, 0.013)	0.0070	(-0.002, 0.016)	-0.0020	(-0.009, 0.006)	0.02	(-0.5, 0.1)
Model 4 [(c)]	*Direct effect*	*Evening Environmental Noise*	0.0050	(-0.002, 0.013)	0.0070	(-0.002, 0.016)	-0.0020	(-0.009, 0.005)	0.02	(-0.05, 0.09)
		Nocturnal Environmental Noise	0.0050	(-0.004, 0.013)	0.0070	(-0.002, 0.016)	-0.0020	(-0.009, 0.006)	0.02	(-0.05, 0.09)

(a) Adjusted for bully/victim propensity (bullying), hair cortisol level, subjective physical activity, psychological well-being as measured by the Kidscreen 27, socioeconomic status, sex and number of stressful events.

(b) Adjusted for victim propensity (bullying), hair cortisol level, subjective physical activity, psychological well-being as measured by the SDQ, socioeconomic status, sex and number of stressful events.

(c) Adjusted for bully/victim propensity (bullying), hair cortisol level, subjective physical activity, psychological well-being as measured by the SDQ, socioeconomic status, sex and number of stressful events.
